# Longitudinal changes in high sensitivity C-reactive protein associated with serum uric acid in the Korean Genome and Epidemiology Study

**DOI:** 10.1038/s41598-023-50951-2

**Published:** 2024-01-03

**Authors:** Anthony Kityo, Sang-Ah Lee

**Affiliations:** 1 Department of Preventive Medicine, School of Medicine, Kangwon National University, Gangwon, 24341 Republic of Korea; 2 Interdisciplinary Graduate Program in Medical Bigdata Convergence, Kangwon National University, Gangwon, 24341 Republic of Korea

**Keywords:** Biomarkers, Medical research, Risk factors, Inflammation

## Abstract

Cross-sectional studies support the role of serum uric acid (SUA) in inflammation, but evidence from cohort studies is scarce. Longitudinal associations between SUA and high-sensitivity C-reactive protein (*hs*-CRP) were examined in the general population. Data for participants from the Health Examinees-Gem cohort (n = 50,028; 40–69 years; 67% women) who were examined between 2004 and 2013 and followed up until 2016 were analyzed. SUA and *hs*-CRP were measured at baseline and during follow-up. SUA was evaluated as a continuous variable and was also divided into sex-specific quartiles. Mean *hs*-CRP levels at follow-up were evaluated using multivariable proportional odds regression, with non-linear smoothed baseline *hs*-CRP levels serving as a covariate. Selected pathological markers were also examined in relation to *hs*-CRP. Increased levels of SUA at baseline were related to increased levels of hs-CRP at follow-up [regression coefficient per mg/dL increase in baseline SUA (*β*) = 0.08, 95% confidence interval (CI), 0.040–0.128]. A dose–response relationship was observed, (*P* for linear trend = 0.0015). The mean values of *hs*-CRP were highest among participants with the highest follow-up but lowest baseline SUA levels. Elevated *hs*-CRP levels at follow up (> 3 mg/L) were positively related to fasting blood glucose levels, triglycerides levels, liver enzymes, and blood pressure, but negatively related to high density lipoprotein cholesterol levels per unit increase in baseline *hs*-CRP. High SUA levels were associated with high *hs*-CRP levels, suggesting a potential role of SUA in inflammation. However, additional research is needed to confirm these findings.

## Introduction

Low-grade, systemic chronic inflammation (SCI) is a significant contributor to the etiology of noncommunicable diseases. Globally, more than half of all deaths are attributed to chronic inflammatory diseases^1^, which underscores the need to identify exposures affecting SCI and therefore SCI-related diseases. C-reactive protein (C-RP) is a biomarker of low-grade SCI^1,2^. Specifically, high-sensitivity C-reactive protein (*hs*-CRP) is the most reliable marker of inflammation in clinical practice^3^. Accordingly, identifying multiple factors related to *hs*-CRP may help to elucidate the underlying determinants of SCI.

Serum uric acid (SUA), is a purine metabolite that has antioxidant effects within normal physiological ranges^4^, but may also exhibit prooxidant characteristics^5–7^. Elevated levels of SUA have been associated with cardiovascular disease (CVD), hypertension, metabolic syndrome, chronic kidney disease (CKD)^8^, cancer^9,10^, and all-cause mortality^11–13^. Several researchers have hypothesized that SUA may induce metabolic abnormalities via inflammatory pathways^14^, including activation of the IkB kinase/IkB alpha/NF-kB^3^, and AMPK (AMP-activated protein kinase)-mTOR (mammalian target of rapamycin) signaling pathways^15^.

However, few population-based studies have evaluated the association between SUA and inflammatory biomarkers such as *hs*-CRP. In a cross-sectional study of 2731 nondiabetic adults, SUA was positively associated with *hs*-CRP after adjusting for age, sex, and body mass index (BMI)^3^. In another cross-sectional study of 1107 individuals, SUA was correlated with serum *hs*-CRP after age and sex adjustment^16^. Raesi et al*.* reported a positive correlation between SUA and *hs*-CRP in 378 Iranian postmenopausal women^17^. SUA was also positively associated with C-RP in 957 community-dwelling elderly participants^18^. Only two longitudinal studies have investigated the association between SUA and *hs*-CRP; one included 3518 Brazilians, and the other included 892 Italians. Both studies reported positive associations between baseline SUA and *hs*-CRP at follow up^19,20^. The only clinical trial on this topic recruited 20 healthy volunteers, and reported that uric acid administration resulted in increased IL-6, but not C-RP^14^.

Most of the previous epidemiological studies on the relationship between SUA and *hs*-CRP have been cross-sectional^3,16,17,19^, and therefore causal conclusions could not be drawn. Moreover, previous studies were limited in sample size^3,16,17,20^, recruited participants with cardiometabolic conditions^3,16^, and adjusted for limited confounders^3,16^. In addition, none of those studies were conducted in an Asian population. To the best of our knowledge, only two longitudinal studies have evaluated the association between SUA and *hs*-CRP^19,20^. More longitudinal studies with large sample sizes taken from diverse populations are needed to determine whether SUA is a potential inflammatory biomarker.

Using a large population study of Korean adults, we evaluated the longitudinal association between baseline SUA and *hs*-CRP levels at follow up. In secondary analyses, the levels of selected pathological markers predicted from changes in *hs*-CRP were evaluated. This study hypothesised that baseline SUA was positively associated with *hs*-CRP at follow-up in individuals without elevated baseline *hs*-CRP (*hs*-CRP > 3 mg/L).

## Materials and methods

### The Health Examinees study

**Figure 1:**
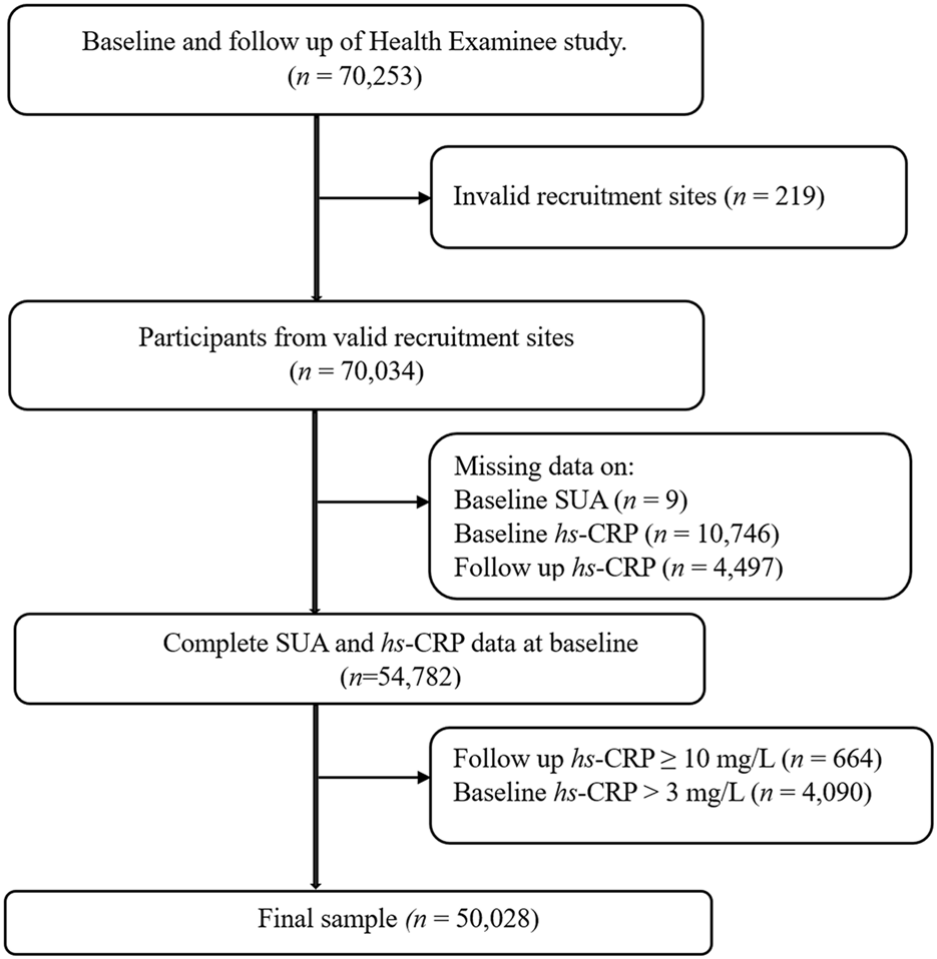
Selection of study participants. *SUA *serum uric acid, *hs*-*CRP* high sensitivity C-reactive protein.

We conducted an observational longitudinal analysis of the Health Examinees study (HEXA) cohort, a prospective population-based substudy within the Korean Genome and Epidemiology study (KoGES) that was established to investigate the etiological factors of complex diseases^21^. The HEXA recruited participants between 2004 and 2013 at 38 health examination centers and training hospitals located in the eight regions of Korea and follow-up studies were conducted between 2007 and 2016. The study details have been published elsewhere^22^. Individuals who participated in both the baseline and follow up surveys of the HEXA study were included in the current analysis (*n* = 70,253, ≥ 40 years). From those individuals, we excluded participants who were recruited from invalid sites: (1) were recruited from sites that participated in the pilot study between 2004 and 2006; (2) were recruited from sites that did not meet the HEXA standards for biospecimen quality control; and (3) were recruited from sites that participated in the study for < 2 years (*n* = 219)^23^. Furthermore, participants who had missing data on baseline SUA (*n* = 9) and *hs*-CRP (*n* = 10,746), those who had missing data on *hs*-CRP at follow-up (*n* = 4497), those with follow-up *hs*-CRP equal to or exceeding 10 mg/L (considered as having acute inflammation, *n* = 664), and those with elevated *hs*-CRP (> 3 mg/L) at baseline (*n* = 4090) were excluded, yielding a final analytical sample of 50,028 participants (Fig. 1).

### Measurement of clinical variables

After an overnight fast, a total sample of at least 19 cc of blood was drawn into a serum separator tube (SST) and two ethylenediamine-tetra-acetic acidtubes and placed into a conical tube for laboratory tests and storage. The biospecimens were given study IDs that were matched to the participants’ questionnaires and labeled with 2D bar code stickers. Biospecimens are kept in refrigerators at each medical institution until they are collected within 24 h by a courier from the commercial laboratory that processes specimens using various laboratory tests^21^. Blood test results were uploaded to the Korean Centers for Disease Control (KCDC) web-based database system. The extracted samples were subsequently transported to and stored at the National Biobank of Korea (https://biobank.nih.go.kr/eng). Baseline SUA, *hs*-CRP, fasting blood glucose (FBG), triglycerides (TG), high-density lipoprotein cholesterol (HDL-C), alanine aminotransferase (ALT), aspartate aminotransferase (AST), creatinine, and gamma glutamyl transferase (GGT) levels were measured using enzymatic calorimetric methods with automatic analyzers (ADVIA 1650 and ADVIA 1800 (Siemens, Tarrytown, NY, USA)^24^. Quality control of biospecimens in the Biobank of Korea has been described in detail elsewhere^25,26^.

### Baseline covariates

Educational level, and household income were assessed in addition to demographic characteristics such as age and sex. Other covariates included lifestyle factors such as: smoking, where current smokers were defined as participants who had smoked more than four hundred cigarettes during their lifetime and were still smoking^27^; drinking categorized as current alcohol drinkers, and past drinkers/never drinkers, where current alcohol drinkers were those who reported that they had ever drunk alcohol and were still drinking alcohol at the time of the interview; and regular physical exercise, assessed by asking participants to report (1) whether they engaged in regular sweat-inducing physical exercise; (2) the number of times they engage in these exercises in a week (1–2 times/week to everyday), and (3) the duration of the exercise. Regular exercise was defined as engaging in activities that caused body sweating for at least five times a week lasting at least 30 min.

Weight and height were objectively measured at baseline by trained medical staff. Body mass index (BMI) was calculated as weight in kilograms divided by the square of height in meters (kg/m^2^). BMI was categorized into four classes based on the WHO classification system of BMI for Asian adults: < 18.5, 18.5–22.9, 23–24.9 and ≥ 25 kg/m^2^^28^. Blood pressure was measured using a mercury sphygmomanometer while the participant assumed a sitting position. Two measurements were used to compute average diastolic (DBP) and systolic (SBP) blood pressure.

The information about diseases and use of medication was obtained using a standardized questionnaire administered by trained staff and self-reported by participants. Diabetes was defined as FBG ≥ 126 mg/dL or drug treatment for elevated FBG, and hypertension was defined as SBP ≥ 130 mmHg or DBP ≥ 85 mmHg or drug treatment for elevated blood pressure. Abdominal obesity was defined as a waist circumference ≥ 90 cm for men and ≥ 80 cm for women^29^. Prevalence of renal function decline was defined using estimated glomerular filtration rate (eGFR < 60 mL/min/1.73 m^2^) that was estimated using the Chronic Kidney Disease Epidemiology Collaboration equation (CKD-EPI)^30^. The number of morbidities was calculated based on the following chronic diseases defined based on self-reported doctor diagnosis and intake of medication: CVD, chronic gastritis, esophageal ulcers, gall bladder disease, fatty liver disease, chronic liver disease, chronic obstructive pulmonary disease, asthma, chronic bronchitis, pulmonary tuberculosis, depression, Parkinson’s disease, arthritis, osteoarthritis, hyper lipidemia, malignant neoplasms, and type 2 diabetes. Based on the number of morbidities, a disease score was created, and participants were categorized as having a disease score of zero (indicating absence of any of the above diseases) or ≥ 1 (indicating presence of any of the above diseases).

### Statistical analysis

Participants were divided into sex-specific quartiles of SUA (Q1–Q4) for descriptive purposes. Participants with missing data on income (> 10%) were categorized as ‘unknown income’, whereas categorical variables with < 5% missing data were replaced by the mode (for education, marital status, smoking, drinking, physical exercise, and metabolic syndrome). Continuous variables with < 5% missing data were replaced by the median. The distribution of participant characteristics according to sex-specific quartiles of SUA was described using percentages for categorical variables or least square means with their standard errors (SE) for continuous variables.

Potential confounders were selected a priori based on previous literature^1,19,20,31^. To minimize potential regression to the mean bias, the change from baseline to follow up was not considered the primary outcome. The main outcome measure was follow up *hs*-CRP levels, adjusted for baseline *hs*-CRP and other covariates including age (spline); sex; marital status (married/cohabiting, single/separated/divorced/widowed/others); (≤ elementary school, middle school, high school, and ≥ university); family monthly income (< 1000, 1000–3000 and ≥ 3000 USD); smoking status (current or past/never smoker); drinking status (current or past/never drinker); regular physical exercise (yes or no); BMI (spline); metabolic syndrome and disease score.

The correlations between follow-up, baseline, and change from baseline to follow up of *hs*-CRP levels according to quartiles of baseline SUA were examined using scatter plots with loess nonparametric smoothers. To examine the longitudinal associations between baseline SUA and *hs*-CRP levels at follow-up, the proportional odds model was fit to estimate the mean values of *hs*-CRP at follow-up as a function of baseline SUA (per mg/dL increment), adjusted for baseline *hs*-CRP and the covariates described above. Smoothed nonlinear effects of baseline *hs*-CRP were fit using restricted cubic splines (RCS) with 4 knots, and allowing the nonlinear effects to differ by quartiles of SUA. Effect modification by sex was tested by including cross-product terms relating to sex and SUA in the model. There was no evidence of an interaction between sex and SUA; thus we did no presentstratified results. In secondary analyses, the odds ratios (ORs) and 95% confidence intervals (CIs) of incident elevation in *hs*-CRP (> 3 mg/L) were calculated using multivariable logistic regression, comparing the highest (Q4) to lowest (Q1) of baseline SUA.

The change in estimated mean *hs*-CRP values at follow-up according to quartiles of follow-up values of SUA, adjusted for baseline SUA levels, was also estimated using the proportional odds model, allowing for nonlinear smoothed effects of baseline SUA. To explore the clinical relevance of *hs*-CRP, the mean values of selected pathological markers (FBG, TG, HDL-C, liver enzymes, and blood pressure) at follow-up were estimated, adjusting for their baseline measurements, and using follow-up *hs*-CRP as the predictor. Baseline values of *hs*-CRP were also included as covariates, and were modeled as RCS to visualize predicted means according to baseline *hs*-CRP.

Descriptive analyses and logistic regression were conducted using SAS software version 9.4 (SAS Institute Inc., Cary, NC, USA), and proportional odds models and graphics were conducted using R software version 4.3.1 (R Foundation for Statistical Computing, Vienna, Austria). P < 0.05 was used to define statistical significance.

### Ethical approval

The study was conducted in accordance with the Declaration of Helsinki, and approved by the Ethics Committee of the Korean Health and Genomic Study of the Korean National Institute of Health and the Institutional Review Boards of all participating hospitals (IRB no. E-1503-103-657).

### Informed consent

Informed consent was obtained from all subjects involved in the study.

## Results

A total of 50,028 participants including 33,593 women, were analyzed. The mean age (mean ± SE) was 53.6 ± 0.07 years. The median values (IQR) of baseline SUA was 4.5 (3.8–5.4) mg/dL and 4.4 mg/dL (3.7–5.0) at follow-up. The median *hs*-CRP at follow up was 0.6 (0.4–1.0) mg/L. After a median (IQR) follow-up of 5.0 (3.9–6.1) years, 2023 (4.0%) participants had elevated *hs*-CRP. Participants who were older, had low education attainment, were current smokers, current drinkers, or who had a high BMI were more likely to have high levels of SUA at baseline (Table 1). Participants with the highest levels of SUA were less likely to engage in regular physical exercise. On the other hand, individuals with high SUA levels were more likely to be diagnosed with metabolic syndrome, and chronic morbidity. Furthermore, *hs*-CRP, liver enzymes and creatinine tended to be elevated among participants in the highest quartile of SUA.

**Table 1:** Baseline characteristics of participants by sex-specific quartiles of serum uric acid.

	Quartiles of serum uric acid			
	Q1 (*n* = 12,584)	Q2 (*n* = 11,534)	Q3 (*n* = 13,984)	Q4 (*n* = 11,926)
Age, years	52.9 ± 0.1	53.1 ± 0.1	53.6 ± 0.1	54.7 ± 0.1
Sex, women, %	67.2	66.1	68.1	67.0
Education level, %				
Elementary	13.9	13.5	14.5	15.2
Middle	15.3	14.4	15.9	16.8
High	41.0	41.0	40.4	40.1
≥ College	29.7	31.1	29.2	27.9
Marital status, married, %	91.5	91.4	90.6	90.0
Income level, %				
Unknown	11.3	11.2	11.0	12.2
< 1	8.9	8.2	8.5	8.8
1–3	37.4	37.2	37.8	38.3
≥ 3	42.4	43.5	42.7	40.7
Current smoker, %	9.2	9.8	9.3	9.6
Current drinker, %	41.1	42.8	43.5	43.4
Regular exercise, %	45.3	43.9	43	42.4
Body mass index, kg/m^2^	23.1 ± 0.02	23.5 ± 0.02	24.0 ± 0.02	24.8 ± 0.02
Metabolic syndrome, %	11.8	13.6	18.2	27.7
CVD, %	2.5	1.9	2.4	3.4
Cancer, %	1.4	1.1	1.2	1.3
Respiratory diseases, %	0.7	0.6	0.5	0.8
GIT diseases, %	1.7	1.4	1.3	1.2
Mental illness, %	0.7	0.8	0.7	0.8
CKD, %	1.9	2.8	3.9	10.3
Joint diseases, %	5	4.6	4.6	5.3
Thyroid disease, %	1.9	1.8	2.1	2.3
Disease score, ≥ 1, %	16.8	16.4	18.0	26.1
SUA, mg/dL				
Men	4.3 (3.8–4.6)	5.2 (5.0–5.4)	6.0 (5.8–6.2)	7.1 (6.7–7.7)
Women	3.2 (2.9–3.4)	3.8 (3.7–3.9)	4.4 (4.2–4.5)	5.2 (5.0–5.6)
*hs*-CRP^a^, mg/L	0.4 (0.3–0.7)	0.5 (0.3–0.8)	0.5 (0.3–0.9)	0.6 (0.3–1.1)
GGT^a^, mg/dL	18.0 (13–25)	19.0 (14–27)	19.0 (15–30)	22.0 (17–37)
AST^a^, mg/dL	21.0 (18–24)	21.0 (18–25)	22.0 (19–26)	23.0 (20–28)
ALT^a^, mg/dL	17.0 (13–23)	18.0 (14–24)	19.0 (15–26)	21.0 (16–29)
*hs*-CRP levels, mg/L, %				
> 3	3.1	3.7	4.2	5.1

Values are %, mean ± SE or median (IQR) unless specified.

*ALT* alanine aminotransferase, *AST* aspartate aminotransferase, *CVD* cardiovascular disease, *CKD* chronic kidney disease, *GIT* gastrointestinal tract, *GGT* gamma glutamyl aminotransferase, *SUA* serum uric acid.

^a^Median (IQR).

There was a strong correlation between baseline and follow up *hs*-CRP by SUA (Figure S1). Furthermore, as baseline *hs*-CRP increased, a greatest reduction in follow-up *hs*-CRP was observed in individuals with lower baseline SUA levels (Figure S1).

The regression coefficients and 95% CIs for *hs*-CRP at follow-up per mg/dL increase in baseline levels of SUA are shown in Table 2. Increased levels of SUA at baseline was related to increased levels of *hs*-CRP at follow-up (β, 0.08; 95% CI 0.040–0.128). The odds ratios and 95% CI for incident elevated *hs*-CRP according to quartiles of SUA are displayed in Table S1. After adjusting for baseline *hs*-CRP and potential confounders, increasing SUA was associated with increased odds of elevated *hs*-CRP [OR (95% CI) for highest vs. lowest SUA levels, 1.21 (1.06–1.38), *P* for trend = 0.010; OR (95% CI) per 1 mg/dL increment in SUA, 1.06 (1.02–1.11)].

**Table 2:** Regression estimates of *hs*-CRP at follow up according to baseline serum uric acid.

	*β*	95% CI	*P* value
Model^a^	0.240	0.18–0.290	< 0.001
Model^b^	0.087	0.040–0.128	0.0015

Regression coefficients and 95% CIs were calculated using proportional odds regression.

^a^Adjusted for age, sex and baseline hs-CRP.

^b^Adjusted for model a, education, income, marital status, alcohol consumption, smoking status, regular physical exercise, BMI (spline), metabolic syndrome, and disease score.

**Figure 2:**
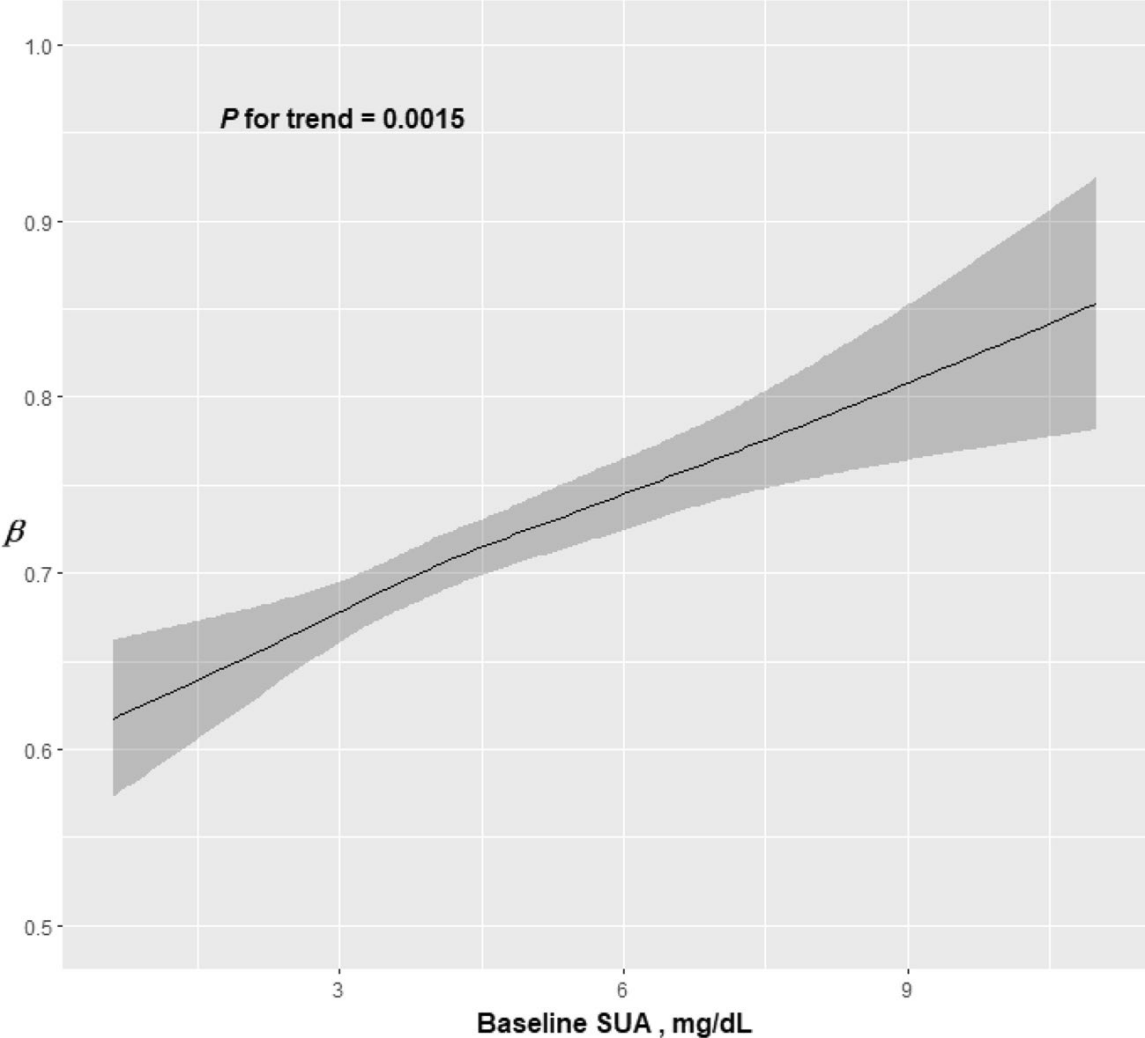
Regression coefficients for *hs*-CRP levels at follow-up according to baseline SUA levels. The dark line represents regression estimates, and bands represent 95% CIs. Coefficients were adjusted for baseline *hs*-CRP, age (spline), sex, education, income, marital status, alcohol consumption, smoking status, regular physical exercise, BMI (spline), metabolic syndrome, and disease score.

The analyses of nonlinear relationships showed a linear increase in follow-up *hs*-CRP levels with increasing baseline levels of SUA (Fig. 2).

**Figure 3:**
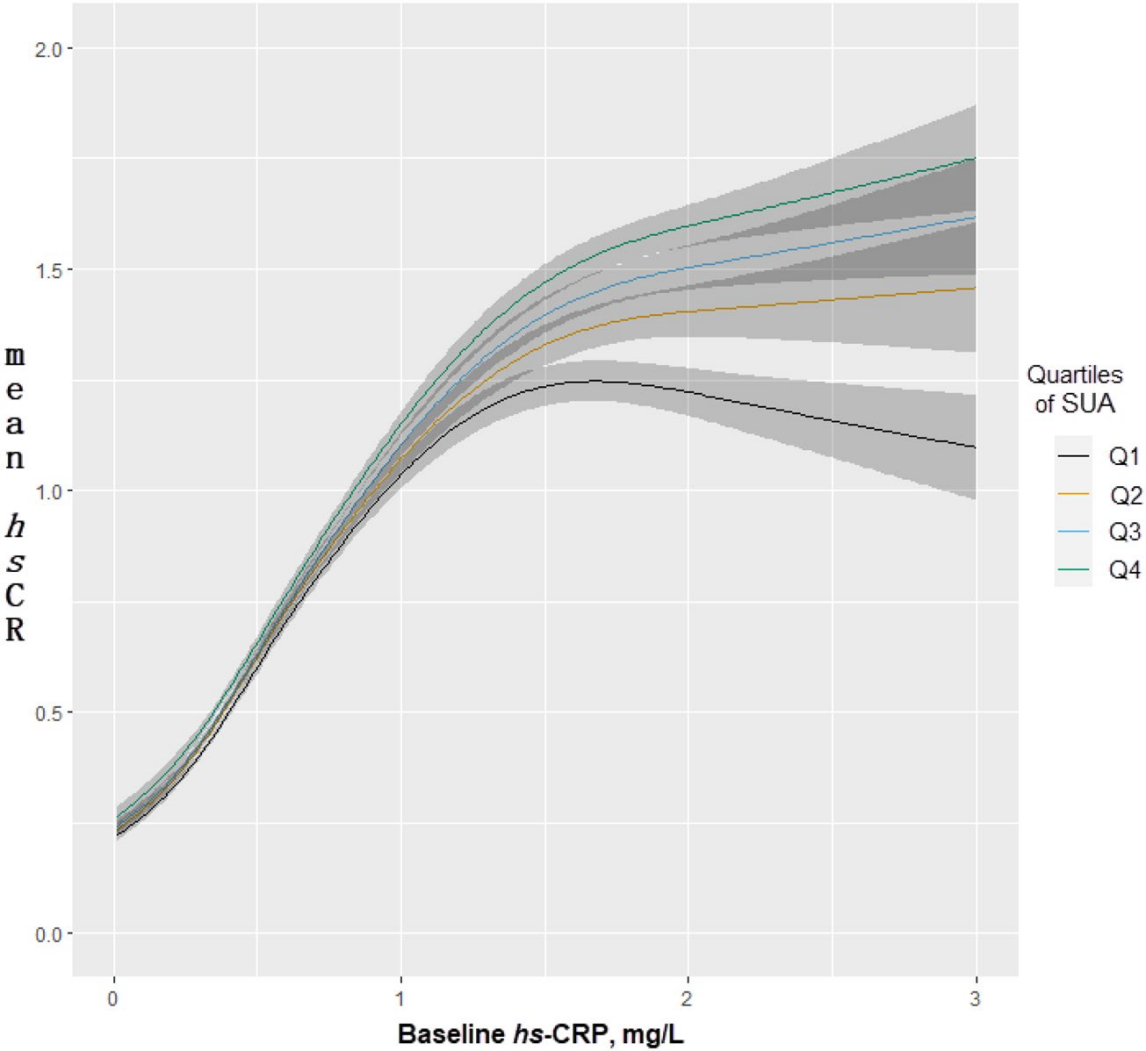
Estimated mean *hs*-CRP at follow up according to levels of SUA at baseline, allowing for non-linear effects of baseline *hs*-CRP. The model was fitted using the proportional odds model, and adjusted for baseline *hs*-CRP, age (spline), sex, education, income, marital status, alcohol consumption, smoking status, regular physical exercise, BMI (spline), metabolic syndrome, and disease score.

The longitudinal relationship between SUA and *hs*-CRP, with smoothed nonlinear effects of baseline *hs*-CRP are displayed in Fig. 3. Improvements in follow-up *hs*-CRP levels were observed with lower baseline SUA among individuals with baseline *hs*-CRP levels > 1.5 mg/L.

**Figure 4:**
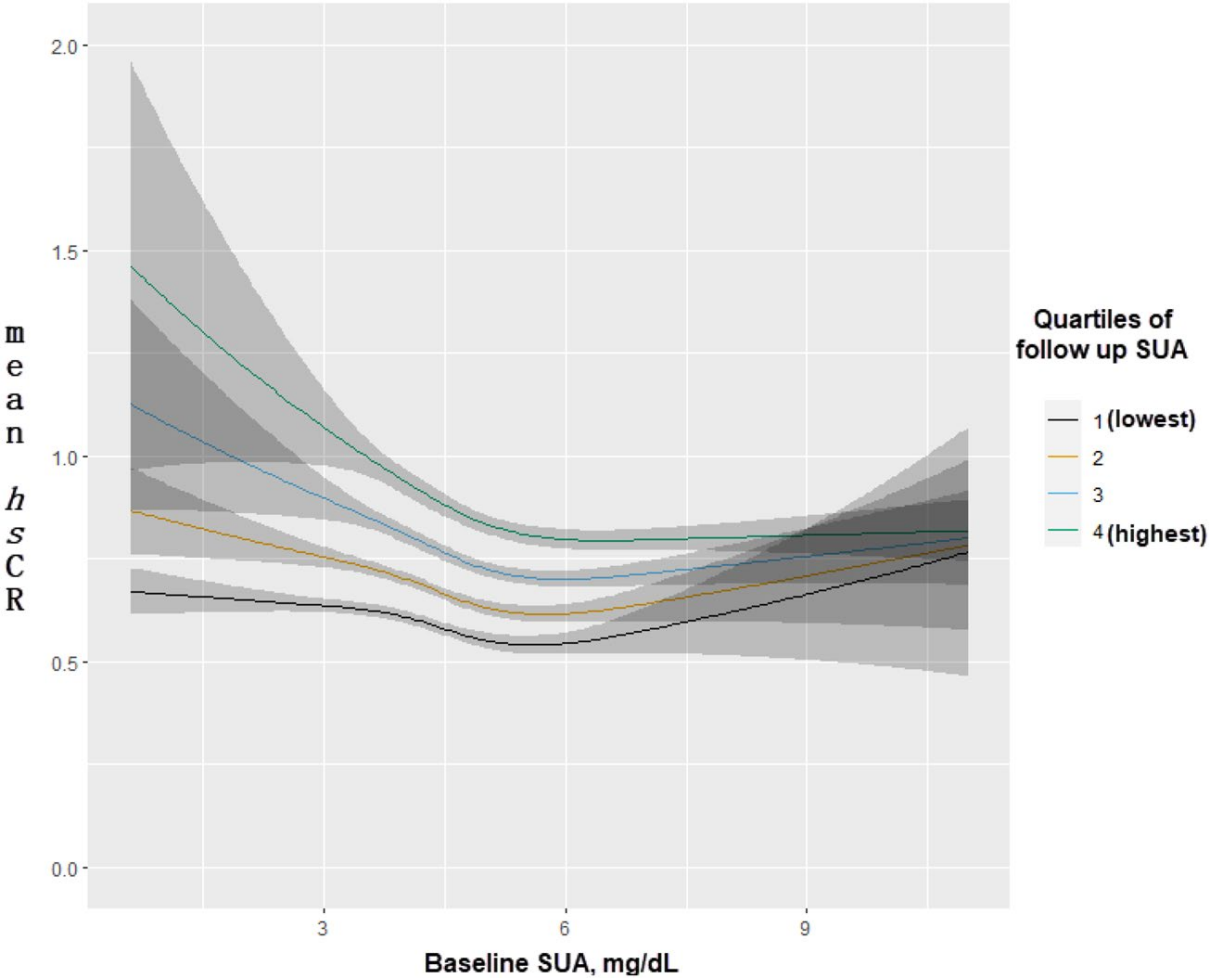
Predicted mean of *hs*-CRP at follow up according to SUA levels at follow up and baseline, with baseline SUA fitted as a smoothed nonlinear covariate, with additional adjustment for baseline *hs*-CRP, age (spline), sex, education, income, marital status, alcohol consumption, smoking status, regular physical exercise, BMI (spline), metabolic syndrome, and disease score.

The predicted mean of *hs*-CRP levels at follow-up according to longitudinal changes in SUA from baseline to follow-up are shown in Fig. 4. Predicted mean values of *hs*-CRP at follow-up were highest among individuals who had the lowest baseline and highest follow-up SUA levels. However, sustained low levels of SUA were associated with the lowest mean values of *hs*-CRP (Fig. 4).

**Figure 5:**
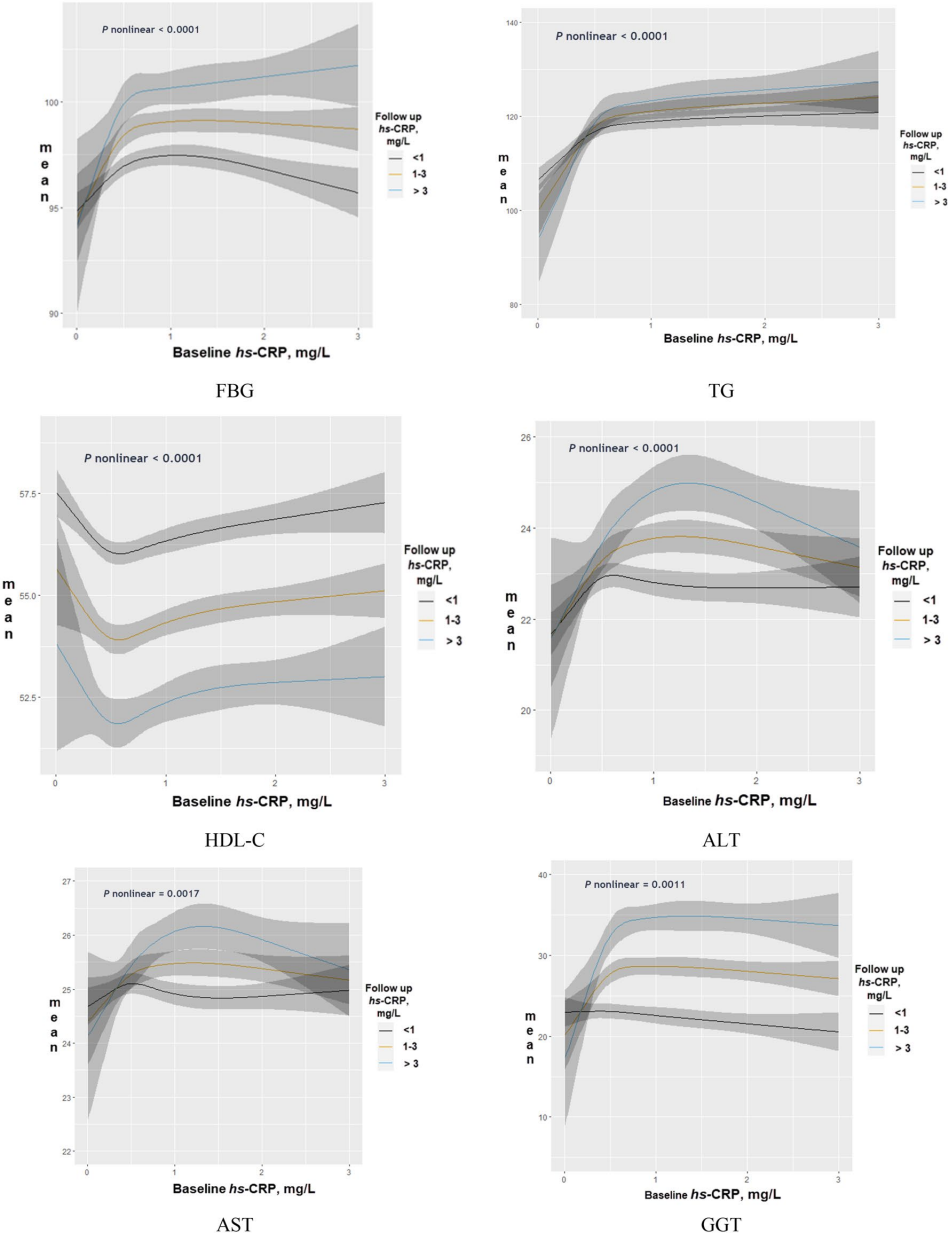
Predicted mean of pathological markers according to longitudinal change in *hs*-CRP, adjusted for baseline pathological markers, *hs*-CRP, age (spline), sex, education, income, marital status, alcohol consumption, smoking status, regular physical exercise, BMI (spline), metabolic syndrome, and disease score.

We examined whether increased *hs*-CRP levels from baseline to follow-up was correlated with pathological markers, and the results are shown in Fig. 5 and Figure S2. Elevated follow-up *hs*-CRP (> 3 mg/L) was associated with the highest mean values of FBG, TG, liver enzymes, blood pressure; and reduced HDL-C levels, particularly at lower levels of baseline *hs*-CRP.

## Discussion

This study used a large population-based cohort of 50,028 Korean adults, and multiple measurements of SUA and *hs*-CRP, to evaluate the longitudinal relationship between SUA and *hs*-CRP after 5 years. Individuals with the highest baseline SUA levels had the highest mean values of *hs*-CRP at follow-up. A linear dose–response relationship between SUA and *hs*-CRP was reported.

To discern the clinical relevance of elevated *hs*-CRP, the predicted means of FBG, TG, HDL-C, liver enzymes, and blood pressure were compared across levels of *hs*-CRP at follow up, adjusted for their baseline values. Serum *hs*-CRP level at follow-up was positively related to FBG, TG, liver enzymes, and blood pressure, and negatively related to HDL-C. The results suggest that SUA may be related to elevated *hs*-CRP levels, indicating SCI in the general Korean population.

The study findings are broadly supported by previous observational studies. Plasma SUA was positively associated with *hs*-CRP in a sample of 2731 outpatient individuals at a general hospital^3^. Raesi et al. reported a positive correlation between SUA and *hs*-CRP in 378 Iranian postmenopausal women^17^. SUA was positively associated with several inflammatory biomarkers including *hs-*CRP in community dwelling elderly participants^18^. In a cross-sectional study of 1107 participants, elevated SUA was related to increased levels of *hs*-CRP^16^. Elevated SUA was also associated with high circulating inflammatory markers including IL-6, CRP and TNF-α, and was negatively associated with IL-1β^32^. Consistent with findings from the present study, two previous longitudinal studies reported that baseline SUA levels predict increased levels of *hs*-CRP, and a high prevalence of elevated *hs*-CRP at follow-up in Brazilian and Italian populations^19,20^. One clinical trial reported null associations between SUA administration and C-RP in 20 volunteers^14^. Clinical trials with relatively large sample sizes are needed to confirm the associations between SUA and *hs*-CRP levels reported in observational studies.

Potential mechanisms have been suggested to explain the relationship between SUA and inflammation. SUA promotes a proinflammatory state by upregulating the production of soluble inflammatory mediators^14^, and increasing the production of reactive oxygen species^33^. SUA was found to exhibit proinflammatory effects by stimulating the expression of several inflammatory biomarkers via activation of the IkB kinase/IkB alpha/NF-kB signaling pathways in hepatic cells^3^. Furthermore, SUA promotes inflammasome-dependent inflammation by regulating the AMPK (AMP-activated protein kinase)-mTOR (mammalian target of rapamycin) signaling pathway^15^. Direct proinflammatory effects of SUA on vascular smooth muscle cells have also been reported^31^.

This study is the first to report on the longitudinal association between SUA and an SCI marker in the general Asian population. These results support the hypothesis that SUA levels may be linked to low-grade SCI, and may serve as a potential marker of SCI if validated with existing inflammatory markers.

Nevertheless, the results from this study should be contextualized within the following limitations. First, only *hs*-CRP was used to evaluate low-grade SCI. Although *hs*-CRP is a marker of inflammation, it is not a specific marker of chronic inflammation, and its optimal clinical use is still debated^33^. Moreover, interindividual variations in *hs*-CRP exist according to ethnicity and age due to genetic polymorphisms and lifestyle factors^33^, suggesting that our findings may not be generalizable to young individuals, or individuals of other ethnicities. In addition, short-term intraindividual variations in *hs*-CRP have been reported. However, we used multiple measures of *hs*-CRP to reduce possible misclassification of individuals resulting from the use of a single measurement^33^. Thus, to validate the role of SUA in inflammation, large population-based cohort studies that use inflammatory scores based on a wide array of inflammatory markers are needed^1^. Second, this was an observational study, which limits inference of causal relationships between SUA and SCI. Nevertheless, we employed a longitudinal modeling approach to minimize reverse causation. Finally, these results may be prone to residual confounding from factors that we could not control for, such as medication since data on prescribed medication could not be accessed. Nevertheless, we adjusted for presence of comorbidities based on self-reported diagnoses and current use of medication for existing chronic diseases.

The major strengths of our study include the use of a large, ethnically homogeneous sample of participants from all regions of Korea-which increases the generalizability of the findings to the entire adult population. The longitudinal design allowed us to examine the temporal relationships between SUA levels and SCI. Adjusting for multiple demographic, lifestyle, and medical-related factors that affect both SUA and *hs*-CRP levels contributed to the reliability of our estimates. Furthermore, the use of biochemical variables that were measured using standardized laboratory procedures, minimized the potential effects of measurement errors in the estimates.

## Conclusion

High SUA levels were independently related to increased levels of *hs*-CRP-suggesting low-grade SCI, in a large population-based sample of Korean adults. This relationship was dose-dependent. Furthermore, high levels of *hs*-CRP positively predicted FBG, TG, liver enzymes and blood pressure, and negatively predicted HDL-C. Future studies are needed to validate SUA using other established inflammatory biomarkers. Nevertheless, these results contribute to further understanding of the role of SUA as a potential proinflammatory marker.

### Supplementary Information

Supplementary Information.

## Data Availability

The dataset used for the analysis in this study is maintained and managed by the Division of Population Health Research at the National Institute of Health, Korea Centers for Disease Control and Prevention. The Health Examinees Study dataset has been merged with the cancer registry data provided by National Cancer Center of Korea in a collaborative agreement. It contains some personal data that may potentially be sensitive to the patients, even though researchers are provided with an anonymized dataset that excludes resident registration numbers. Other researchers may request access to the data by contacting the following individuals at the Division of Population Health Research, National Institute of Health, Korea Centers for Disease Control and Prevention: Senior Staff Scientist Dr. Jung Hyun Lee (jaylee1485@korea.kr); Director Dr. Kyoungho Lee (khlee3789@korea.kr).
