# Supplementary material for: Longitudinal changes in high sensitivity C-reactive protein associated with serum uric acid in the Korean Genome and Epidemiology Study
Source: Sci Rep. 2024 Jan 3;14:374. doi: 10.1038/s41598-023-50951-2 (PMC10764782; doi:10.1038/s41598-023-50951-2)
Supplement: Supplementary file 1 — Supplementary Information. [file 41598_2023_50951_MOESM1_ESM.docx]

**Longitudinal Changes in High sensitivity C-reactive Protein Associated with Serum Uric Acid in the Korean Genome and Epidemiology Study**

Anthony Kityo , Sang-Ah Lee

Table S1. Odds ratios and 95% CI of elevated *hs*-CRP (*hs-*CRP >3mg/L) at follow-up according to baseline serum uric acid

|  | Quartiles of serum uric acid | | | |  |  | Per 1mg/dL |
| --- | --- | --- | --- | --- | --- | --- | --- |
|  | Q1 | Q2 | Q3 | Q4 | *P* for trend |  |  |
| *hs*-CRP > 3mg/L, n | 394 | 514 | 507 | 608 |  |  | 2023 |
| OR (95% CI)^1^ | 1.00 | 1.12 (0.97-1.29) | 1.21 (1.06-1.38) | 1.26 (1.10-1.44) | 0.001 |  | 1.10 (1.04-1.16) |
|  |  |  |  |  |  |  |  |
| OR (95% CI)^2^ | 1.00 | 1.13 (0.98-1.29) | 1.22 (1.06-1.39) | 1.27 (1.11-1.44) | 0.001 |  | 1.10 (1.05-1.16) |
|  |  |  |  |  |  |  |  |
| OR (95% CI)^3^ | 1.00 | 1.12 (0.97-1.28) | 1.19 (1.04-1.36) | 1.21 (1.06-1.38) | 0.01 |  | 1.06 (1.02-1.11) |

Odds ratios and 95% confidence intervals were computed by multivariable logistic regression

^1^ Adjusted for age and sex and baseline *hs*-CRP.

^2^ Adjusted for age, sex, education, income, marital status, drinking, smoking, exercise

^3^ Adjusted for age, sex, education, income, marital status, drinking, smoking, exercise, BMI, metabolic syndrome, and comorbidity

| 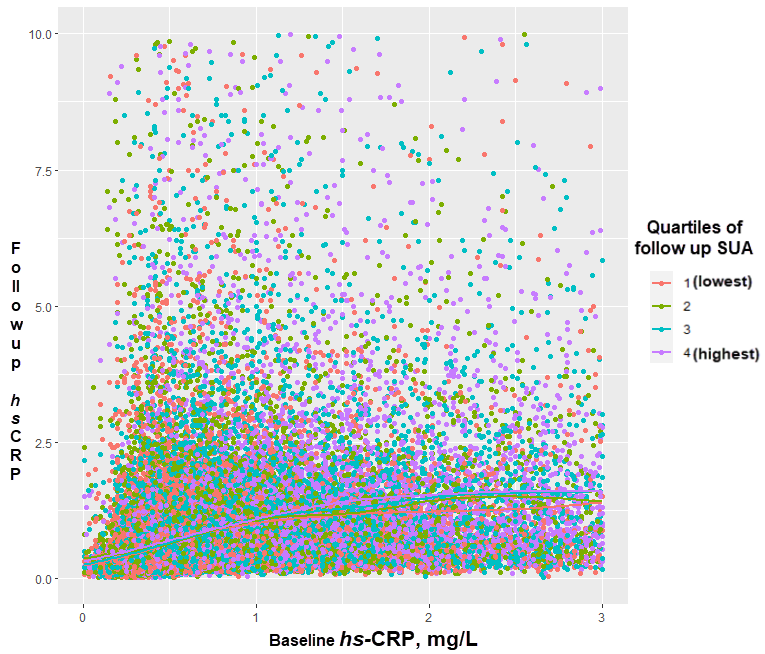 | 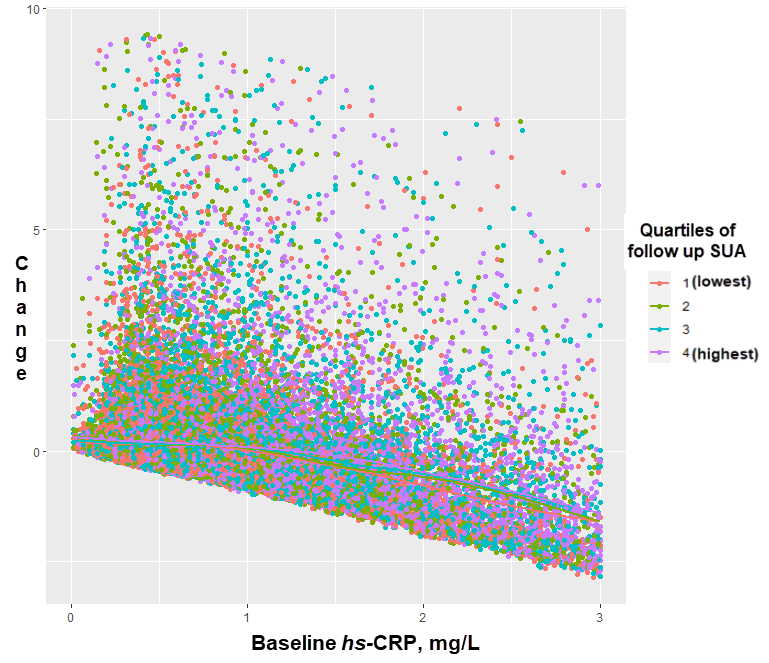 |
| --- | --- |
| A | B |

Figure S1. Correlation of follow up and baseline *hs*-CRP (A) and changed from baseline to follow up *hs*-CRP by baseline SUA. Lines are nonparametric loess smoothers

| 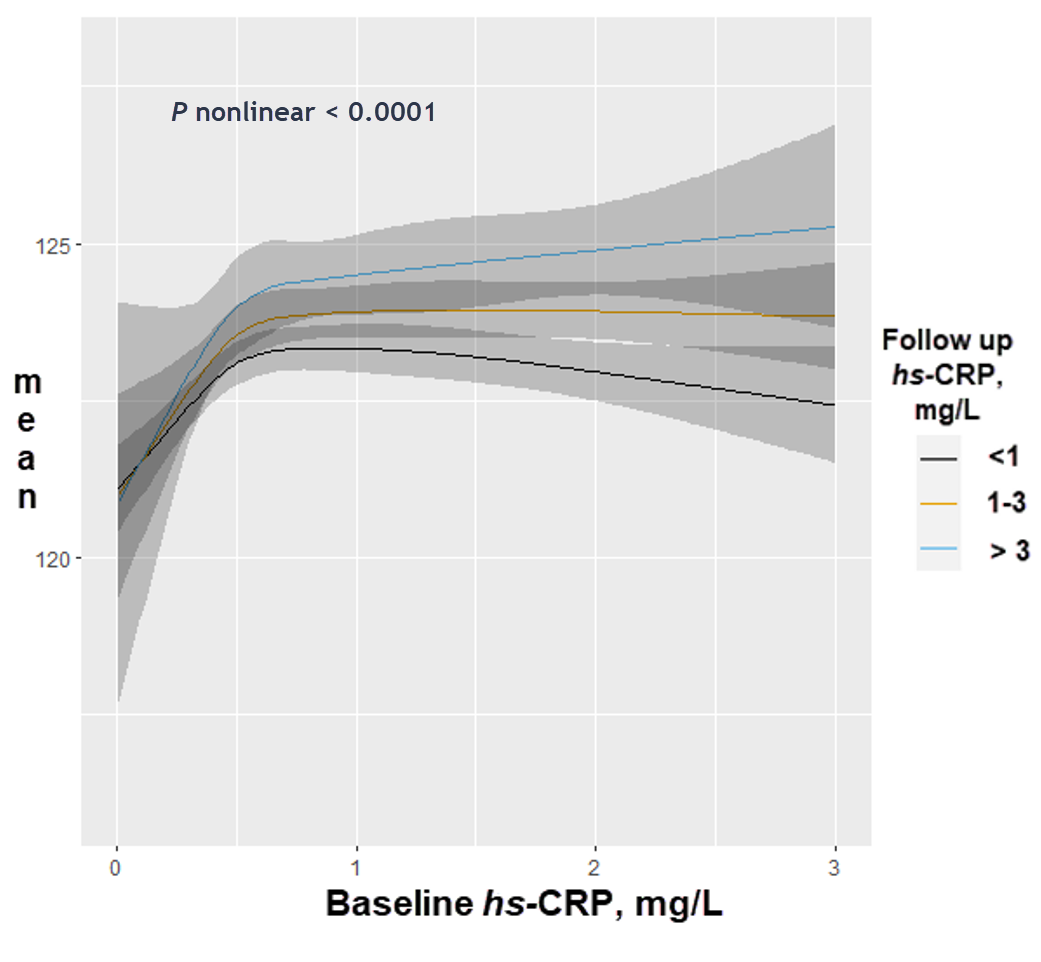 | 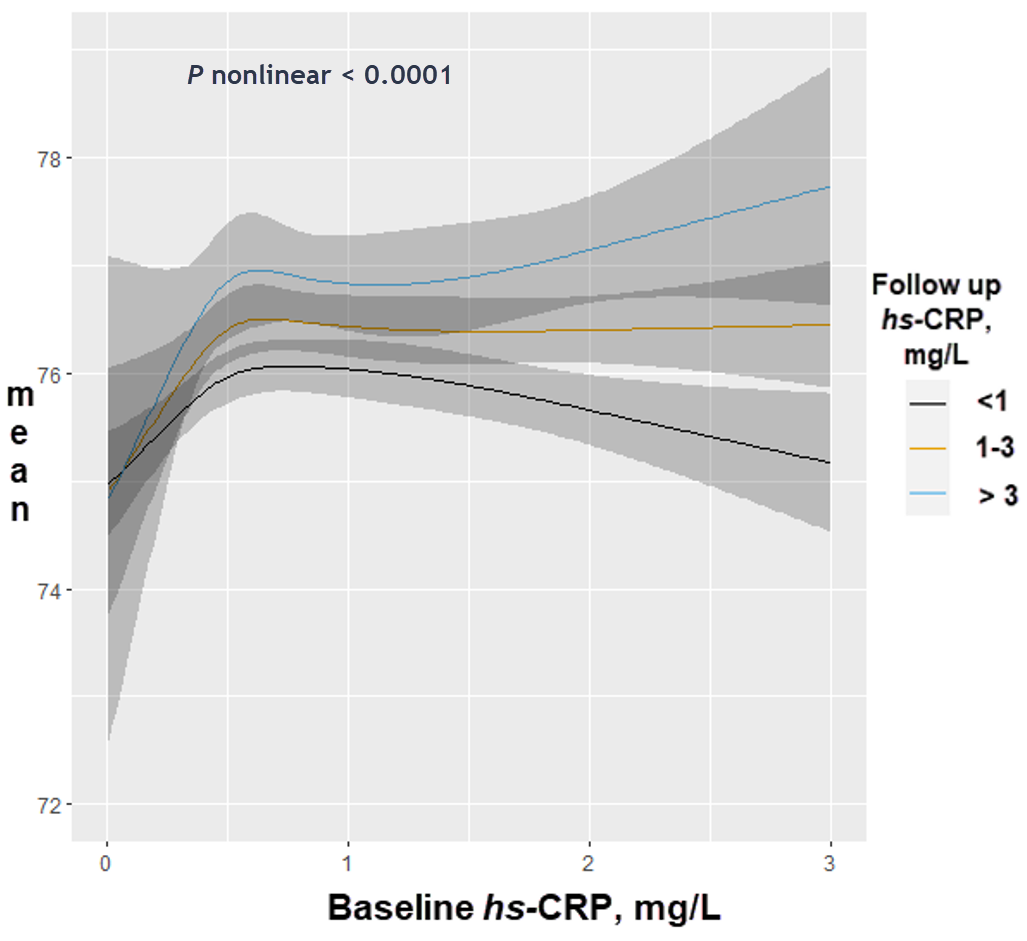 |
| --- | --- |
| Systolic blood pressure | Diastolic blood pressure |

Figure S2. Predicted means of follow up blood pressure measurements according to longitudinal change in *hs*-CRP, adjusted for baseline blood pressure measurements, *hs*-CRP, age (spline), sex, education, income, marital status, drinking, smoking, regular physical exercise, BMI (spline), metabolic syndrome, and comorbidity score.
